# Supplementary material for: Identification of photoperiod-induced specific miRNAs in the adrenal glands of Sunite sheep (Ovis aries)
Source: Front Vet Sci. 2022 Jul 22;9:888207. doi: 10.3389/fvets.2022.888207 (PMC9354845; doi:10.3389/fvets.2022.888207)
Supplement: Supplementary file 1 [file Data_Sheet_1.ZIP › Supplementary file/miRDeep2 file.zip!/miRDeep2 file/Know_Structure/SP42A2/oar-mir-133.pdf]

miRBase precursor : oar-mir-133
Total read count : 1041
oar-miR-133 read count : 1014
remaining reads : 27

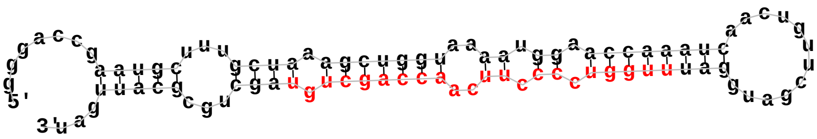

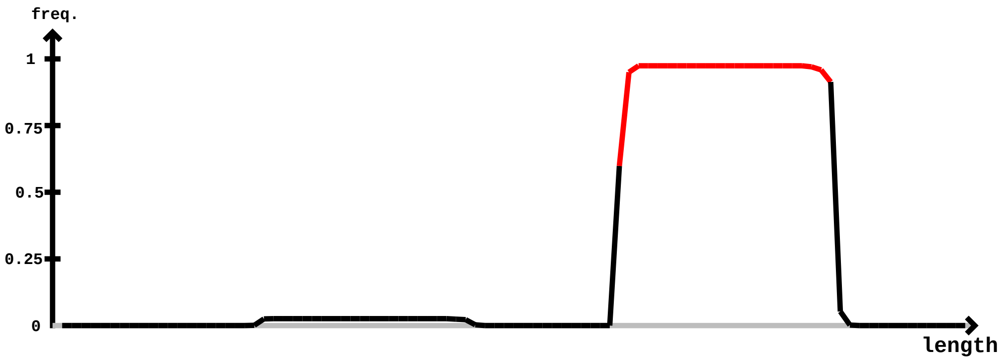

oar-miR-133

```
5'- gggaccgaaugcuuugcuaaagcugguaaaauggaaccaaaucaacuguucgauggauuugguccccuucaaccagcuguagcugcgcauugau   -3'    exp
    .......(((((...((((.(((((((..((.((.((((((((............)))))))).)).))..))))))).))))...)))))...   reads      mm         sample
    ...................Gagcugguaaaauggaaccaa......................................................   1          1          seq
    ....................agcugguaaaauggaaccaa......................................................   1          0          seq
    ....................agcugguaaaauggaaccaaa.....................................................   2          0          seq
    ....................agcugguaaaauggaaccaaau....................................................   19         0          seq
    ....................agcugguaaaauggaaccaaauc...................................................   3          0          seq
    .....................gcugguaaaauggaaccaaau....................................................   1          0          seq
    .........................................................uuugguccccuucaaccagc.................   4          0          seq
    .........................................................uuugguccccuucaaccagcu................   12         0          seq
    .........................................................uuugguccccuucaaccagcuA...............   8          1          seq
    .........................................................uuugguccccuucaaccagcug...............   35         0          seq
    .........................................................uuugguccccuucaaccagcugu..............   545        0          seq
    .........................................................uuugguccccuucaaccagcugua.............   19         0          seq
    ..........................................................uugguccccuucaaccagcug...............   4          0          seq
    ..........................................................uugguccccuucaaccagcugu..............   330        0          seq
    ..........................................................uugguccccuucaaccagcugua.............   31         0          seq
    ..........................................................uugguccccuucaaccagcuguag............   1          0          seq
    ...........................................................ugguccccuucaaccagcugu..............   21         0          seq
    ...........................................................ugguccccuucaaccagcugua.............   3          0          seq
    ...........................................................ugguccccuucaaccagcuguag............   1          0          seq
```
